# Eligibility for the use of ready-made spectacles among children in a school-based programme in Ghana

**DOI:** 10.1371/journal.pgph.0000079

**Published:** 2022-01-27

**Authors:** Frederick Afum Asare, Priya Morjaria

**Affiliations:** Department of Clinical Research, London School of Hygiene and Tropical Medicine, London, United Kingdom; University of Zimbabwe, ZIMBABWE

## Abstract

Ready-made spectacles are low-cost spectacles for correcting refractive errors in children who would otherwise have their refractive errors uncorrected due to lack of availability and affordability of conventional, expensive custom-made spectacles. Thus, this study seeks to estimate the proportion of children with uncorrected refractive errors eligible for ready-made spectacles in a school-based programme. A school-based descriptive cross-sectional study was employed to screen children aged 12–15 years in eighteen public junior high schools within the Bongo district of Ghana. Children who failed the 6/9 acuity test were refracted and given spectacles. Ready-made spectacle was prescribed when visual acuity improved by ≥2 lines in at least one eye with full correction (astigmatism of ≤0.75D); spherical equivalent corrected visual acuity to ≤1 line worse than best corrected visual acuity with full correction in the better eye; and there was ≤1.00D difference between the two eyes. A total of 1,705 school children were examined. Of this number, 30 (1.8%; 95% CI: 1.2–2.5%) met the criteria for refractive correction but none had any. Twenty-six (86.7%; 95% CI: 69.7–95.3%) were found to be eligible for ready-made spectacles (power range: -1.50D to +1.00D, mean spherical equivalent ± SD = -0.27D ± 0.79D) while 4 (13.3%; 95% CI: 4.7–30.3%) were not, hence, given custom-made spectacles. A binary logistic regression analysis revealed that the odds of being eligible for one type of spectacles was similar between males and females (OR: 1.1; 95% CI: 0.1–12.7; p = 0.93). A large proportion of students who met the criteria for spectacle correction could be corrected with ready-made spectacles. There is, therefore, the need for these spectacles to be considered an appropriate alternative for refractive error correction during school eye health programmes.

## Introduction

Uncorrected refractive error remains the leading cause of visual impairment in children in both developed [1, 2] and developing countries [3–6]. In 2004, it was reported to affect about 13 million children aged 5–15 years globally [7]. A recent publication by the Lancet Global Health Commission on Global Eye Health has indicated that in 2020, there was an estimated 1.44 million blind (including uncorrected refractive error) children aged 0–14 years, 22.16 million with moderate to severe vision impairment and 46.60 million with mild vision impairment globally [8].

The most common treatment for refractive error is with spectacles. Even though it is a simple and straightforward intervention, the high costs, non-availability, and poor access to refractive services in many settings [9–15], limits their use as an appropriate correction which results in many people often living with different levels of visual impairment. Spectacle correction is crucial in mitigating the immediate and long-term consequences a child with uncorrected refractive error will have, such as poor academic performance, decreased quality of life, decreased social development and loss of employment opportunities [7]. As such, school-based screening and spectacle distribution programmes which provide spectacles (both custom and ready-made spectacles) have become increasingly common in recent years [16] to address this problem in a cost-effective manner.

Ready-made spectacles are a part of this cost-effective solution. They are low-cost quality spectacles (USD 2.42 per patient) [17] which are widely used in school eye health screening programmes. Where appropriate, after ensuring that a child meets the strict criteria for dispensing, they provide a spherical correction in each eye and can be dispensed on the spot [18]. They can also be produced in large quantities and are dispensed immediately and quickly in schools or clinics. Even so, a drawback to their use is that they require a large inventory of frames in different sizes, colours, and shapes, with each having a range of power lenses and are only suitable if the maximum spherical equivalent power is ±3.50 dioptres [19].

However, owing to the new design and range of spectacles, the ‘Ready-to-Clip’ spectacles, children with uncorrected refractive errors of varying degrees are able to get corrected because these spectacles have lenses that are interchangeable between the right and left eyes and are clipped into the person’s chosen frame according to their individual prescription [19]. Thus, they enable the provision of spectacles for children with a difference in prescription in their two eyes which is less than or equal to 1.00 dioptre [19]. It has also been found that they can be dispensed for spherical equivalent of up to ±6.00 dioptres as reported in one of the studies which used a variant of it [20].

Despite the unequivocal importance and benefits of ready-made spectacles in correcting refractive error in children, and their tendency to solve a supply chain problem which is prevalent in Africa, most of the studies conducted on these spectacles and their benefits in school children have mainly been in Asia, particularly China and India with none in Africa. For instance, a study by Zhu et al. on Chinese school children revealed that ready-made spectacles could substantially alleviate visual morbidity in two-thirds or more of visually impaired school children in China [21]. Another by Zeng et al. in China reported that the use of ready-made spectacles is suitable in school-based refractive services programmes as it saves costs and improves the logistics of service delivery [22]. Similarly, in a non-inferiority randomised clinical trial by Morjaria et al. in India, it was found that ready-made spectacles could reduce costs for school-based eye health programmes in India without compromising spectacle wear as about 86% of children with uncorrected refractive error were eligible for ready-made spectacles, and the proportion that was wearing ready-made spectacles (75.5%) was not inferior to that wearing custom-made spectacles (73.6%) at 3 to 4 months follow up [23]. In addition to their significant cost-saving potential which stands at about 58.6% cost-saving per child needing spectacles of USD 15.76 [24], it has also been found that 70–90% of children with uncorrected refractive errors could potentially benefit from ready-made spectacles [22, 23, 25] making it an appropriate alternative for refractive error correction in regions/countries like Ghana where supply of custom-made spectacles is problematic due to the purported high average cost of GHC 150 (USD 30) of prescription glasses.

This study investigated the proportion of school children with uncorrected refractive errors in the Bongo District of the Upper East Region of Ghana that are eligible for ready-made spectacles to address the adherence to spectacle wear due to the lack of accessibility and affordability. It also provides valuable evidence for the use of ready-made spectacles as an alternative in correcting refractive errors in children as there is no data available in the region and in the country at large.

## Methods

### Study design

The study was a school-based descriptive cross-sectional study in the Bongo district of Ghana. We used a multistage random sampling technique to recruit school children aged 12–15 years in public junior high schools within the district.

### Sampling

A list of all public junior high schools within the Bongo District was obtained from the statistical unit of the Ghana Education Service. These schools were stratified according to the six sub-districts within the district and in each sub-district, three schools were randomly selected by balloting. All school children aged 12–15 years within each randomly selected school were then screened.

### Inclusion, eligibility, and exclusion criteria

The following inclusion and eligibility criteria, which were adapted from a randomised clinical trial on ready-made spectacles and custom-made spectacles by Morjaria et al. [26] in 2016 was used. They included all school children aged 12–15 years in public junior high schools, presenting visual acuity (that is, with spectacles if usually worn) of less than 6/9 in either eye, visual acuity with full correction that improves by two or more lines in at least one eye, cylindrical power of not more than 0.75 dioptre of the full correction, spherical equivalent (that is, the sum of the myopic or hyperopic correction in dioptres plus half the astigmatic cylindrical correction) which corrects visual acuity equal to, or not more than one line worse than best corrected visual acuity with full correction in the better eye and a difference in spherical equivalent between the two eyes of not more than 1.00 dioptre. Children whose visual acuity did not improve adequately with the spherical equivalent, with other causes of vision loss, and with anisometropia (more than 1.00 dioptre difference between the two eyes) were excluded and referred for further examination in the district or regional eye hospital.

### Sample size determination

The sample size was calculated with the formula [27]:

$$s=\frac{x^{2}np(1-p)}{d^{2}(n-1)+x^{2}p(1-p)}\mathrm{where},$$

*s* = required sample size, *x*^*2*^ *=* the table value of chi-squared for 1 degree of freedom at the desired confidence level (3.841), *n* = population size (~5000), *p* = population proportion (assumed to be 0.50 since this would provide the maximum sample size), *d* = degree of accuracy expressed as a proportion (0.05). Based on these parameters, a sample size of 357 was calculated, which was multiplied by a design effect of 2.5 to account for clustering. A non-response rate of 10% was then applied and the final sample size required was 982.

### Ethical consideration

Ethical approval was obtained from the Ethics Committee at the London School of Hygiene and Tropical Medicine and the Navrongo Health Research Centre Institutional Review Board in the Upper East Region of Ghana. Permission from the district director of the Ghana Education Service, written informed consent from head teachers at each participating school and written informed consent from parents of participating students were also obtained. Assent was further obtained from the students themselves and documented accordingly after a verbal explanation of the whole study was given to them. Participation was free and voluntary, and the study adhered to the tenets of the Declaration of Helsinki.

### Data collection procedure and examination

Nurses were recruited to conduct the vision assessment while a data entry person completed the data collection forms at the schools. A pilot study was conducted in one selected school (which was not part of the schools for the main study). A two-day training was then held by the principal investigator (FA) for the team to assess the robustness of the data collection form and the level of accuracy of visual acuity measurement by the nurses. All ambiguous items were then modified before the start of the actual study.

**Fig 1:**
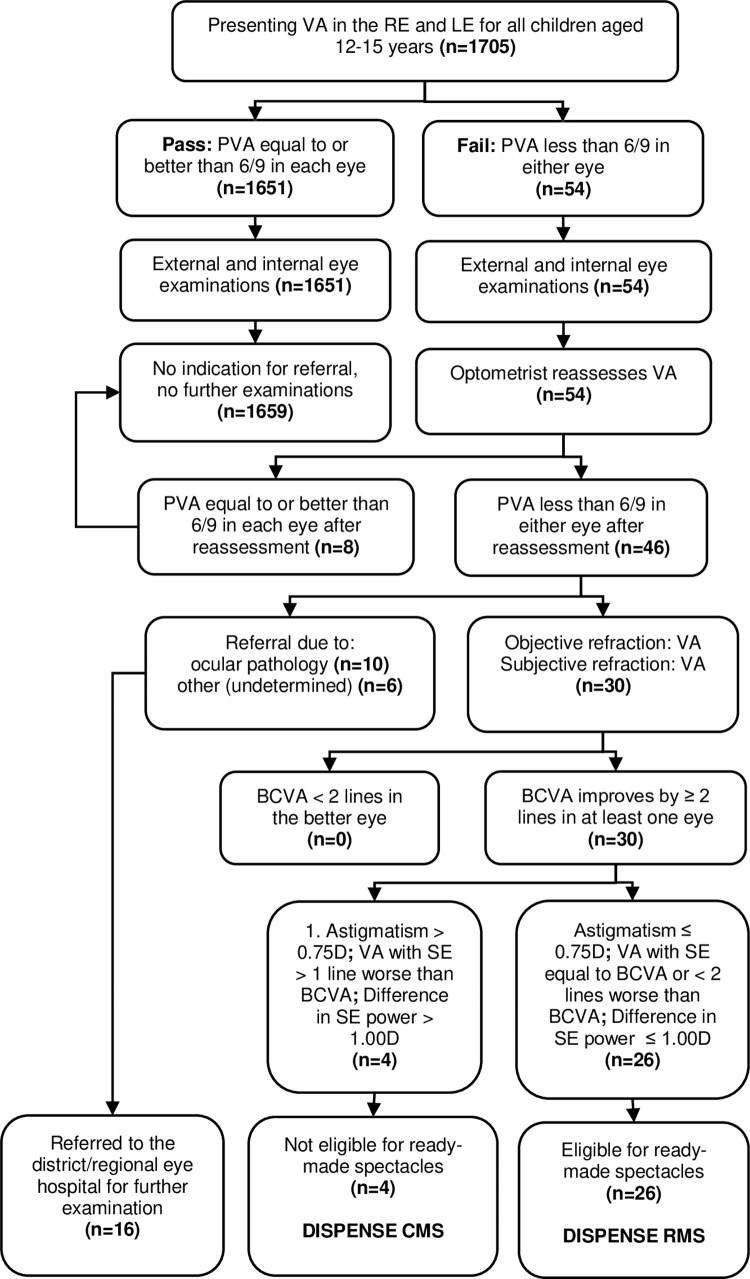
Examination procedures for enlisting participants for ready-made or custom-made spectacles. **Abbreviations:** n = number; VA = visual acuity; RE = right eye; LE = left eye; PVA = presenting visual acuity; BCVA = best corrected visual acuity; D = dioptre; SE = spherical equivalent; CMS = custom-made spectacles; RMS = ready-made spectacles.

The following procedures were followed for data collection during the study:

1. Data on students’ demographics were first collected and unique identification was assigned to each participant.
2. Presenting visual acuity of each student was then measured by the trained nurses with the Snellen visual acuity chart at 6 meters. This was done for the right and left eyes, respectively.
3. Students with presenting visual acuity of 6/9 or better in each eye with more than 2 lines reduction on the +1.00-blur test were said to have no refractive error, hence no further examination. However, students who failed to have reduction with the +1.00 test were referred for further examination and refraction while those who failed the 6/9 visual acuity test in either eye were referred to the optometrist for reassessment of vision and refraction.
4. Non-cycloplegic objective refraction with a retinoscope (Riester ri-scope RI.10543-slit HL, 2.5V), subjective refraction and best corrected visual acuity in each eye were then measured.
5. Spherical equivalent for each eye was calculated and the best visual acuity it provided in each eye was recorded.
6. Students whose visual acuity improved with full correction by two or more lines in the better seeing eye and had a spherical equivalent which did not reduce vision to more than one line worse than the best corrected visual acuity in the better eye, were eligible for ready-made spectacles. Other criteria that certified one as eligible for ready-made spectacles were astigmatism of less than or equal to 0.75 dioptre and a difference in spherical equivalent between the two eyes of less than or equal to 1.00 dioptre.
7. Those who were eligible for the ready-made spectacles were given on the spot, while those who were not received custom-made spectacles a week after the refraction was done. This is because the spectacles were sent to the dispensing laboratory in the capital city for glazing and fixing, which took some days. Both types of spectacles were provided at no cost.
8. Time taken to complete the entire refraction (objective and subjective) and dispensing of the spectacles was also recorded using a stopwatch. These times were then compared between the dispensing of the ready-made spectacles as against the custom-made spectacles.
9. External and internal eye examinations were conducted with a head loupe and an ophthalmoscope (Welch Allyn REF, 11710, NY, USA) to detect other pathologies by the qualified optometrist. Fig 1 illustrates the examination procedure for the study.

### Statistical analysis

Data collected from the study were input in Microsoft Excel and exported to STATA 15.1 (StataCorp, College Station, TX, USA) for analysis. The data were analysed as frequency, percentages, and proportions. Mean, standard deviations, prevalence, and 95% confidence intervals were also estimated for quantitative variables with a significance level of 0.05 was used. A binary logistic regression was then used to investigate the relationship between gender and the type of spectacles dispensed. The results of the statistical analysis were then presented in the form of tables and graphs.

## Results

### Study population

A total of 1,705 (94.0%) children out of 1,817 school children in eighteen public junior high schools were screened/examined. More than half (55.4%) of the children examined were females, with their ages ranging from 12 to 15 years. The mean age ± (SD) was 14.1 ± (0.9) years. All children were classified as living in rural settings. Table 1 summarises the characteristics of participants.

**Table 1:** Characteristics of participants screened.

Characteristics	Frequency (%)
**Participants**	
Underwent screening	1705 (93.8)
Not screened (absent from school)	112 (6.2)
**Gender**	
Male	760 (44.6)
Female	945 (55.4)
**Age**	
12	59 (3.5)
13	395 (23.1)
14	620 (36.4)
15	631 (37.0)
**Study area**	
Rural	1705 (100.0)
**Participants screened/sub-district**	
Central	318 (18.7)
Namoo	207 (12.1)
Soe	306 (17.9)
Beo	290 (17.0)
Zorko	282 (16.5)
Vea	302 (17.7)

### Inclusion and exclusion for spectacle correction

Out of the total number of children who were screened, 46 (2.7%; 95% CI: 2.0–3.6%) were unable to read the 6/9 optotypes. Of this number, 30 (65.2%) met the criteria for spectacle correction after refraction revealed they had uncorrected refractive errors (that is, their presenting visual acuity improved with full correction by two or more lines in the better seeing eye). The rest (34.8%) were however, excluded from the spectacles assessment and referred due to ocular pathology (21.8%) and other undetermined causes (13.0%). Table 2 summarises the various pathologies detected which excluded students from spectacle assessment.

**Table 2:** Ocular factors for exclusion from spectacle correction and referral.

Ocular factors for exclusion	Male n (%)	Female n (%)	Total n (%)
Macular scar	1 (6.3)	2 (12.5)	3 (18.8)
Traumatic cataract	0 (0.0)	1 (6.3)	1 (6.3)
Corneal opacity	0 (0.0)	2 (12.5)	2 (12.5)
Retinitis pigmentosa	0 (0.0)	1 (6.3)	1 (6.3)
Severe vernal keratoconjunctivitis	1 (6.3)	1 (6.3)	2 (12.5)
Known glaucoma	1 (6.3)	0 (6.3)	1 (6.3)
Other (undetermined)	3 (18.8)	3 (18.8)	6 (37.5)
Total	6 (37.5)	10 (62.5)	16 (100.0)

### Distribution of ready-made and custom-made spectacles

Of the 30 (1.8%; 95% CI: 1.2–2.5%) students who underwent assessment for spectacles, 26 (86.7%; 95% CI: 69.7–95.3%) were eligible for ready-made while the rest (13.3%; 95% CI: 4.7–30.3%) were not, hence were given custom-made spectacles (Table 3). About one-fifth (19.2%) of those who were eligible for ready-made spectacles had an unequal refractive power of ≤1.00 dioptre in their two eyes, while the majority (80.8%) of them had isometropia (equal refractive power in the two eyes). However, none of the students who had refractive error had spectacle correction. Even though there were more females who were eligible for both ready-made and custom-made spectacles than males, this was not statistically significant (X^2^ = 0.007, *df* = 1, p = 0.93). A binary logistic regression analysis further revealed that the odds of females being eligible for one type of spectacle was the same as that for males (OR: 1.1; 95% CI: 0.1–12.7; p = 0.93).

**Table 3:** Distribution of ready-made and custom-made spectacles.

Characteristics	Ready-made spectacles		Custom-made spectacles		
Age (in years)	Male n (%)	Female n (%)	Male n (%)	Female n (%)	Total n (%)
12	0 (0.0)	1 (3.3)	0 (0.0)	0 (0.0)	1 (3.3)
13	2 (6.7)	5 (16.7)	0 (0.0)	2 (6.7)	9 (30.0)
14	1 (3.3)	9 (30.0)	1 (3.3)	1 (3.3)	12 (40.0)
15	3 (10.0)	5 (16.7)	0 (0.0)	0 (0.0)	8 (26.7)
Total	6 (20.0)	20 (66.7)	1 (3.3)	3 (10.0)	30 (100.0)

### Spherical equivalent power dispensed

**Fig 2:**
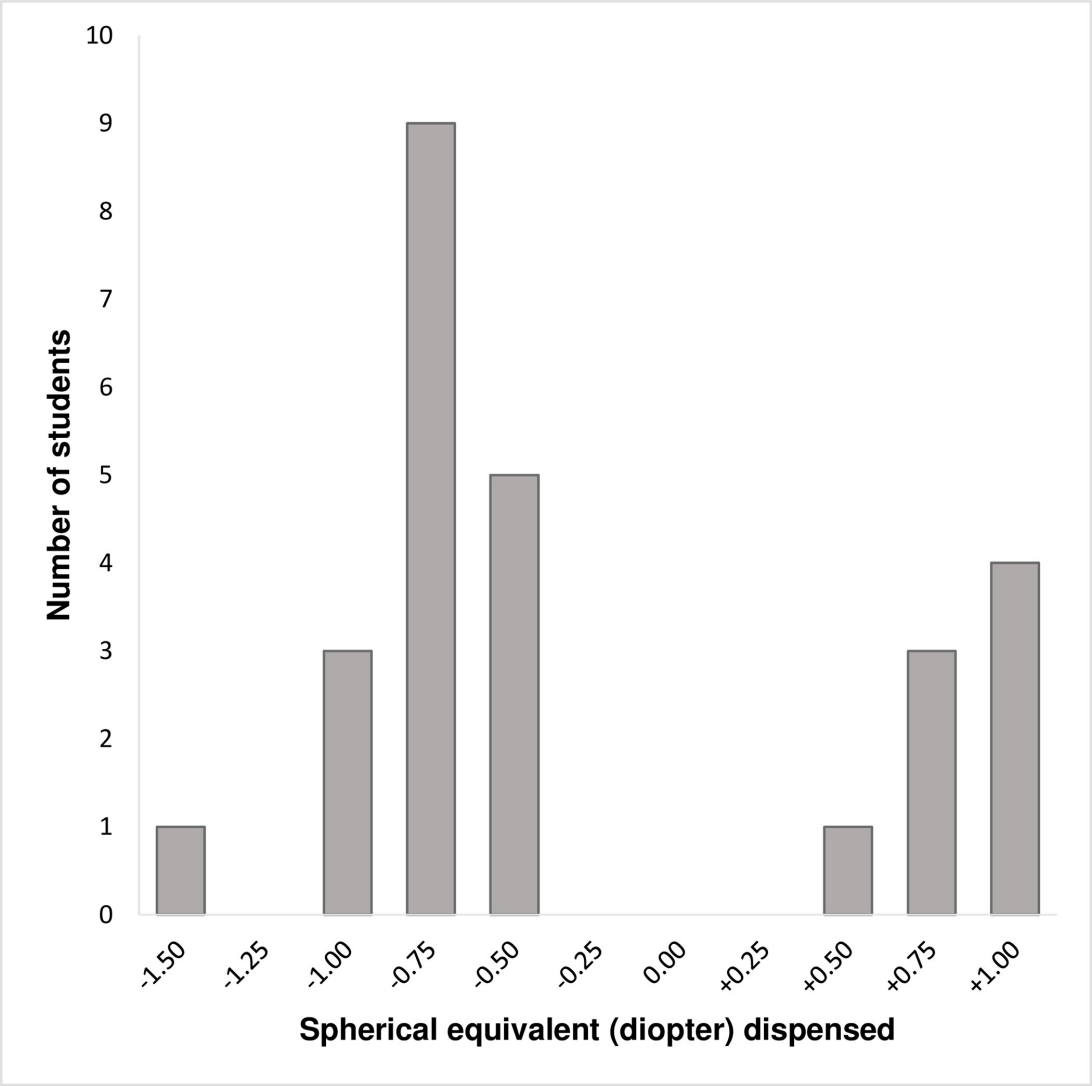
Range of spherical equivalent power dispensed.

The mean spherical equivalent ± (SD) in the better seeing eye dispensed to all children who received ready-made spectacles was -0.27 ± (0.79) dioptre and ranged from -1.50 dioptres to +1.00 dioptre. Of these prescriptions, -0.75 dioptre was the most dispensed while +0.50 and -1.50 dioptres was the least dispensed. Fig 2 describes the range of spectacles dispensed.

## Discussion

This was a school-based descriptive cross-sectional study in Ghana to assess the proportion of students with uncorrected refractive errors that can benefit from ready-made spectacles and to the best of the authors’ knowledge, this is the first study on ready-made spectacles (specifically, ready-to-clip spectacles) among school children in Ghana. It will therefore serve as the basis for further studies on the use of ready-made spectacles in school eye health programmes.

Of the 30 students who met the criteria for spectacle correction, approximately 87% were eligible for ready-made spectacles. Although, as earlier indicated, no studies have been done on ready-made and custom-made spectacles among children in Ghana or Africa, evidence from studies in other parts of the world indicates that about 70–90% of children with uncorrected refractive errors could benefit from ready-made spectacles [22, 23, 25]. Thus, the proportion observed in this study is consistent with that reported in those studies [22, 23, 25].

It was also observed that there was no statistically significant difference in the eligibility for ready-made spectacles between female students and their male counterparts. The odds of females being eligible for ready-made spectacles compared to males were 1:1, which suggests that both males and females with refractive errors will equally benefit from ready-made spectacles once they are identified to be eligible for them.

The spherical equivalent power dispensed (powers of ready-made spectacles) were all available on the spot, which enabled students who were eligible for them get them immediately after refraction. This study used the ‘ready-to-clip’ ready-made spectacles [19], hence, students with an unequal refractive power in their two eyes (≤1.00 dioptre) also benefited. For instance, of those who were eligible for ready-made spectacles, five had an unequal refractive power in their two eyes while the rest had isometropia. However, all these students received their spectacles on the spot.

With respect to the time taken to dispense ready-made spectacles as against custom-made spectacles, it was found that the time taken to dispense spectacles is dependent on the type of spectacles dispensed. The mean time ± standard error of the mean (SEM) (28.0 ± (0.9) minutes; range: 22–38 minutes) taken to dispense ready-made spectacles was slightly more than that taken to dispense custom-made spectacles (21.5 ± (1.4) minutes; range: 18–25 minutes) due to the additional time in calculating the spherical equivalent and conducting subjective refraction with the calculated power. Nonetheless, when the time taken to order, glaze and deliver custom-made spectacles are factored, they take longer to dispense. Thus, the authors believe that the benefits of dispensing ready-made spectacles for the correction of refractive error in children are still significant since they are readily available (dispensed on the spot), accessible and affordable (less costly than conventional custom-made spectacles).

### Strengths

One of the key strengths of this study lies in the fact that ready-to-clip spectacles were used, which provided the opportunity to dispense for children with unequal refractive power (≤ 1.00D) in their two eyes compared to earlier versions of ready-made spectacles which only provided correction for refractive errors with equal powers in both eyes. Another was that a large proportion of students with uncorrected refractive errors were eligible for ready-made spectacles and can be mandated in school eye health programmes. Finally, the use of different frame designs in the study afforded the school children the opportunity to select frames of their choice which could enhance spectacle wear.

### Limitations

A limitation was that a larger sample would have been ideal to provide a greater number of students who will be eligible for the ready-made spectacles. However, a total sample size of 1,705 was able to provide an appreciable number of eligible students for analysis.

Refractive errors among school children were determined and classified using non-cycloplegic refraction as observed in other studies [11, 28, 29]. Even though cycloplegic refraction would have been ideal as it would identify latent hyperopia, especially in children with high amplitude of accommodation, this was not used to prevent blurry vision from the cycloplegic agent which might have interfered with the academic activities of the children. In addition, instillation of cyclopentolate is considered an invasive procedure, hence, for ethical reasons, parental permission would have been challenging due to several cultural beliefs in the district concerning the instillation of eyedrops in children’s eyes during research studies. As such, a non-cycloplegic procedure was used to enable many parents to enrol their children in the study.

## Conclusion

A large proportion of students who met the criteria for spectacle correction could be corrected with ready-made spectacles. There is, therefore, the need for these spectacles to be considered an alternative to refractive error correction in regions like Ghana where there is a challenge with the supply chain of spectacles. However, in order to achieve this, appropriate measures need to be in place to ensure testing at regular intervals for those children that have already been identified with a refractive error and those that are entering school for the first time. A consistent supply at an affordable price for these spectacles is also something that need to be built in to any school eye health programme. Further studies across the country to investigate the proportion of school children in different age groups that will be eligible for ready-made spectacles may also be beneficial to inform public health approach necessary for the correction of refractive error through school eye health programmes.

## Supporting information

S1 Table Full refractions of participants eligible for ready-made and custom-made spectacles. (DOCX)
