# Supplementary material for: Eligibility for the use of ready-made spectacles among children in a school-based programme in Ghana
Source: PLOS Glob Public Health. 2022 Jan 27;2(1):e0000079. doi: 10.1371/journal.pgph.0000079 (PMC10021990; doi:10.1371/journal.pgph.0000079)
Supplement: S1 Table — (DOCX) [file pgph.0000079.s001.docx]

**S1 Table: Full refractions of participants eligible for ready-made and custom-made spectacles**

| **ID** | **Right eye** | | **Left eye** | |
| --- | --- | --- | --- | --- |
| **Ready-made spectacles** | | | | |
| 001 | -0.50 | | -0.75 | |
| 002 | -0.75 | | -0.75 | |
| 003 | -0.50/-0.50 x180 | | -0.50/-0.50 x 180 | |
| 004 | +0.75 | | +0.75 | |
| 005 | -0.75/-0.50 x 180 | | -0.50/-0.50 x 180 | |
| 006 | -1.50 | | -1.50 | |
| 007 | -0.50 | | -0.50 | |
| 008 | -1.00 | | -1.00 | |
| 009 | +1.25/-0.50 x 90 | | +1.50/-0.50 x 90 | |
| 010 | -0.75 | | -0.75 | |
| 011 | -0.50 | | -0.50 | |
| 012 | -0.75 | | -0.50 | |
| 013 | +0.75 | | +0.75 | |
| 014 | -1.00 | | -0.75 | |
| 015 | -0.50/-0.50 x180 | | -0.50/-0.50 x180 | |
| 016 | -0.50 | | -0.50 | |
| 017 | +1.00 | | +1.00 | |
| 018 | +1.00 | | +1.00 | |
| 019 | -0.50/-0.50 x 180 | | -0.50/-0.50 x 180 | |
| 020 | +1.00 | | +1.00 | |
| 021 | -0.75 | | -0.75 | |
| 022 | -1.00 | | -1.00 | |
| 023 | +0.75 | | +0.75 | |
| 024 | +0.75/-0.50 x 180 | | +0.75/-0.50 x 180 | |
| 025 | -0.75 | | -0.75 | |
| 026 | -1.00 | | -1.00 | |
| **Custom-made spectacles** | | | | |
| 001 | +0.75/-1.00 x 180 | | +0.75/-1.00 x 180 | |
| 002 | -1.00/-0.75 x 60 | | -1.00/-0.75 x 150 | |
| 003 | -0.75/-0.75 x 180 | | -0.50/-0.75 x 180 | |
| 004 | Plano/-0.75 x 170 | | Plano/-0.75 x 15 | |
|  | |  | |  |
